# Signal or noise? RNA-binding proteins and the challenges of binding site assignments

**DOI:** 10.1093/nar/gkag452

**Published:** 2026-05-14

**Authors:** Sudeep Sahadevan, Thileepan Sekaran, Matthias W Hentze, Joel I Perez-Perri, Thomas Schwarzl

**Affiliations:** European Molecular Biology Laboratory (EMBL), Meyerhofstraße 1, 69117 Heidelberg, Germany; European Molecular Biology Laboratory (EMBL), Meyerhofstraße 1, 69117 Heidelberg, Germany; European Molecular Biology Laboratory (EMBL), Meyerhofstraße 1, 69117 Heidelberg, Germany; Gulbenkian Institute for Molecular Medicine (GIMM), Edifício Egas Moniz, Avenida Professor Egas Moniz, 1649-028 Lisbon, Portugal; European Molecular Biology Laboratory (EMBL), Meyerhofstraße 1, 69117 Heidelberg, Germany

## Abstract

A significant body of research has been devoted to pinpointing and cataloguing the binding sites of RNA-binding proteins (RBPs) on target transcripts. The most common techniques involve crosslinking and immunoprecipitation (CLIP) followed by high-throughput sequencing. In this review, we provide a comprehensive summary of the major advancements in CLIP-based techniques and state-of-the-art data analysis methods designed for identifying and analysing the binding sites of RBPs. We also brief on methods used to determine the functional relevance of these binding sites and, in addition, delve into the major hurdles faced in the detection and elucidation of the binding sites of RBPs. Finally, we explore reproducibility concerns in the CLIP field, and conclude by suggesting potential avenues for future improvements.

## Graphical Abstract

**Graphical Abstract:**
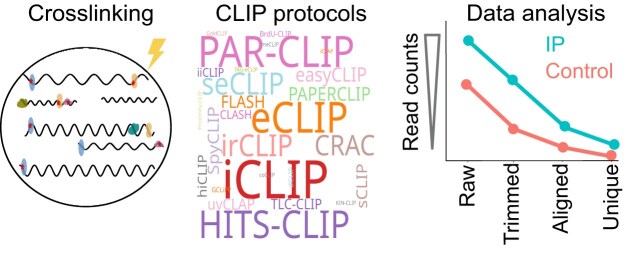

## Introduction

RNA-binding proteins (RBPs) are key players in co- and post-transcriptional regulation such as pre-mRNA splicing, messenger RNA (mRNA) localization, degradation, and translation [1, 2]. They are thus vital for maintaining cellular homeostasis. Dysfunction of RBPs can lead to a plethora of diseases including neurological disorders, cardiovascular diseases, or cancer [3–6]. Recent evidence suggests that mammalian cells could bear >2000 RBPs. Among these, more than half lack discernible RNA-binding domains (RBD) or known RNA-related functions, and thus are referred to as “non-canonical” [7]. While canonical RBPs tend to bind to a well-characterized primary sequence and/or a secondary structure, many non-canonical RBPs do not exhibit such binding preferences for their target RNAs [4, 8–12]. In the cellular milieu, an RNA molecule can interact with multiple RBPs, and an RBP can interact with multiple RNAs. The affinity of RBPs for distinct RNAs influences this interplay, directing interactions toward specific RNA-binding sites. These interactions are also shaped by the concentrations of proteins and RNAs, competition among RNAs for binding to the same protein, and competition among proteins for binding to the same RNA region. The interactions between RBPs and RNAs are mediated by determinants such as specific RNA moieties, binding pockets, Van der Waals (VdW) interactions, hydrogen bonds, hydrophobic, and π interactions [13]. Although VdW interactions and hydrogen bonds tend to exhibit similar amino acid preferences, the estimated ratios of VdW to hydrogen bonds vary. Generally, VdW interactions are considered to be more dominant [13]. Hydrophobic interactions occur between RNA bases and hydrophobic side chains whereas π interactions are facilitated by nitrogenous base rings and π-containing amino acids (phenylalanine, tyrosine, and tryptophan). Both hydrophobic and π interactions contribute to the overall stability of protein–RNA complexes [13].

Upon binding, RBPs can regulate target RNA fate by either enhancing or preventing the recruitment of protein complexes, or by influencing transcript condensation. Conversely, bound RNAs can also control the functions of RBPs, a process collectively referred to as riboregulation. Through riboregulation, RNAs can modulate protein functions such as oligomerization [14], enzymatic activity [15–17], protein–protein interactions [18], complex formation [19] as well as gene network properties [20]. RBPs can also functionally interact with other RBPs, at least in two different modes: cooperative and competitive. In cooperative mode, two or more RBPs bind closely or distantly to an RNA and regulate it synergistically [13, 21–23], resulting in a different outcome compared to a single RBP binding to the same target. In competitive mode, the binding of an RBP to an RNA antagonizes the binding of another, competing RBP often with opposing regulatory consequences [13, 21, 22].

Major advancements in both experimental and bioinformatic techniques to study RBPs have enabled the research community to study the properties of an RBP and the bound RNA in detail. Nevertheless, these advancements have also introduced certain challenges into the field. In this review, we discuss the major experimental and bioinformatic advancements for studying the RNAs associated with a specific RBP and brief on the major challenges faced by both experimental and data analysis methods. We conclude by considering future directions for the optimization of binding site detection.

## Whodunit of RNA–protein interactions: characterizing RNAs binding to an RBP

**Figure 1.**
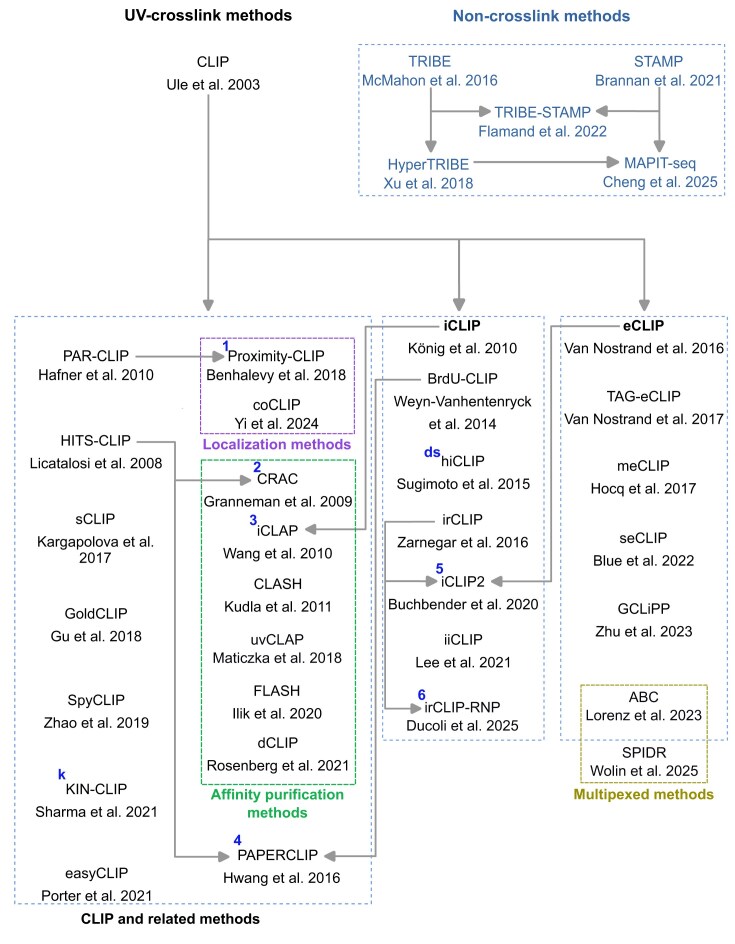
Chronological overview of CLIP and noncrosslink methods. Arrows between the methods are used to indicate the relationship between them. Some CLIP methods are further organized into specialized sub-groups such as localization methods, affinity purification methods and multiplexed methods. Superscript in blue letters at the upper left corner indicates additional notes that are described below. Numbered superscripts indicate protocols that incorporate techniques or improvements from other protocols while alphabetical superscripts denote specialized protocols. **1**: Proximity-CLIP combines the PAR-CLIP protocol with the APEX2-mediated proximity biotinylation technique to study RNA–RBP localization [30]. **2**: CRAC protocol is similar to HITS-CLIP, with the exception of using protein tags and stringent affinity purification under denaturing conditions [31]. **3**: iCLAP closely follows the iCLIP protocol but incorporates a two-step affinity purification process under denaturing conditions [32]. **4**: PAPER-CLIP adopts sample preparation, IP, sodium dodecyl sulfate–polyacrylamide gel electrophoresis (SDS–PAGE) and RNA extraction steps from HITS-CLIP and sequencing library construction step from BrdU-CLIP [33]. **5**: iCLIP2 incorporates improvements such as complementary DNA (cDNA) pre-amplification before size selection (as in irCLIP), and the adoption of independent adapter ligation and cDNA purification steps (as in eCLIP) [34]. **6**: irCLIP-RNP combines irCLIP with mass spectrometry to identify proteins co-bound to RNA with an RBP of interest [35]. **ds**: hiCLIP is designed for identifying RNA duplexes binding to RBPs [36]. **k**: KIN-CLIP is used to study binding kinetics of RBPs and associated RNAs [37].

Methods to study interactions between RBPs and RNAs can be categorized into two classes: RNA-centric methods, to discover proteins that bind to specific RNAs of interest, and protein-centric methods, to determine the RNAs that bind to a particular RBP. In this review, we focus on the latter. Numerous protein-centric methods exploit the property that proteins in direct (“zero length”) contact with RNAs can be covalently crosslinked to those RNAs in their native environment by UV light [24–26]. While formaldehyde (chemical) crosslinking has also been used to study RNA–protein interactions, this method presents notable drawbacks: formaldehyde crosslinking induces protein–protein crosslinks in addition to the desired protein–RNA crosslinks [27, 28]. Moreover, formaldehyde crosslinks are reversible, which can lead to material loss during the protocol. Considering these limitations, we will focus on protein–RNA crosslinking using UV irradiation. Crosslinking and immunoprecipitation (CLIP) was first employed to characterize RNAs bound to Nova proteins around 20 years ago [29]. Since then, crosslinking followed by RNA purification and high-throughput sequencing has become one of the most widely used methods for studying RNA interactors of RBPs. Figure 1 shows a chronological overview of the major CLIP-related protocols published to date.

**Figure 2.**
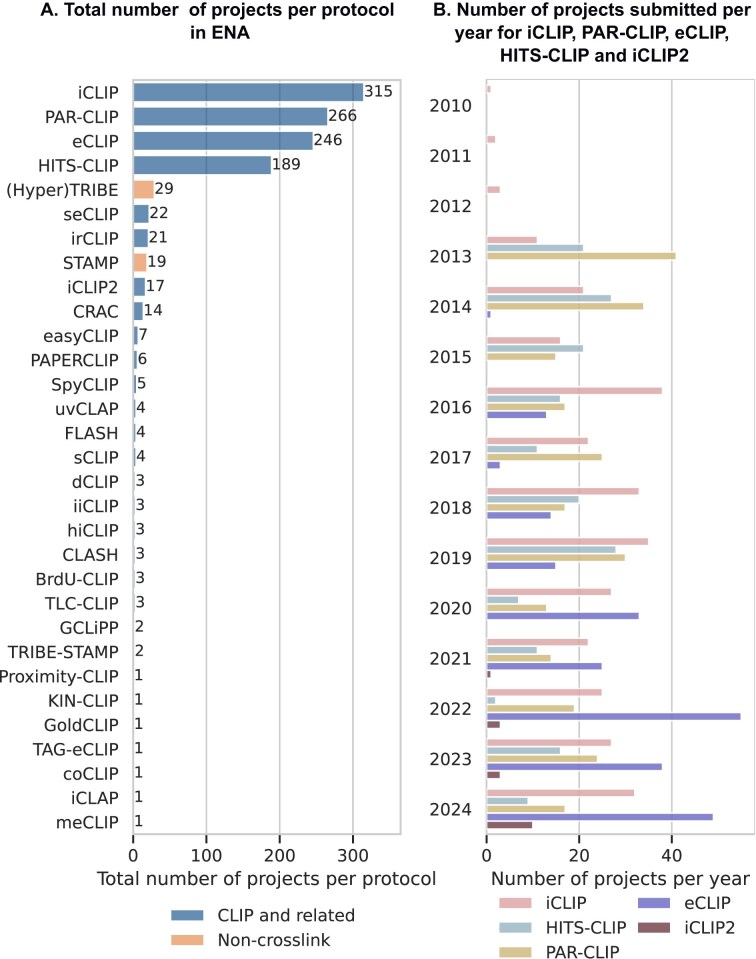
CLIP protocol usage. (**A**) Number of projects per protocol submitted to European Nucleotide Archive (ENA) during the period 2007 - 2024. Project identifiers were retrieved by a keyword search using the EBI Search RESTful API. Blue bars represent the number of projects per protocol for CLIP and related methods, while orange bars represent the number of projects per protocol for non-crosslink methods. (**B**) Number of projects submitted per-year for top four protocols (iCLIP, PAR-CLIP, eCLIP, and HITS-CLIP) and iCLIP2 (improved iCLIP variant).

To assess the extent to which the different CLIP protocols are used within the research community, we used the number of projects submitted to the European Nucleotide Archive (ENA) per protocol over a 17-year period (2007–2024) (Fig. 2A). For each protocol, we retrieved the unique project identifier, title and abstract of associated projects by performing keyword searches using the EBI Search RESTful API. We then used the unique project identifiers to tabulate the total number of projects for each protocol. Our assessment revealed that the top four protocols in terms of the number of submitted projects are: iCLIP [38], PAR-CLIP [39], eCLIP [40], and HITS-CLIP [41]. Combined, these four protocols account for 86% of the total ENA projects surveyed (Fig. 2A). Figure 2B shows the number of projects submitted per year for these four methods along with iCLIP2 (an improved iCLIP variant) [34]. A general trend observed is that the number of iCLIP project submissions has remained relatively similar over the years, whereas submissions for PAR-CLIP and HITS-CLIP fluctuate. In contrast, the number of eCLIP and iCLIP2 project submissions has shown a year-to-year increase.

### CLIP: the state-of-the-art knows a plethora of variations on a theme

High-throughput sequencing of RNA isolated by CLIP (HITS-CLIP), originally applied to study Nova proteins, is one of the first protocols coupling CLIP with microarrays and/or high-throughput pyrosequencing for identifying RNAs bound by an RBP of interest [41]. Using high-throughput sequencing techniques, HITS-CLIP maps RBP footprints with a resolution of ~30–60 nucleotides [42, 43]. Processive reverse transcriptases used in CLIP protocols can read through the crosslinked peptide–RNA adducts, introducing mutations (with 8%–20% frequency), due to the interference of the crosslink with normal Watson–Crick base pairing [43–45]. This property was leveraged for the analysis of crosslink-induced mutation sites (CIMS), thus increasing the resolution of HITS-CLIP data for the crosslink site to the single nucleotide level [43]. In photoactivatable ribonucleoside-enhanced CLIP (PAR-CLIP), cells are incubated with a photoactivatable ribonucleoside analog (such as 4-thiouridine, 4SU), which is then stochastically incorporated into newly synthesized RNAs [46]. 4SU crosslinks to interacting proteins when cells are irradiated with long-wavelength UV (360 nm). During reverse transcription, dG is incorporated opposite to the crosslinked photoadduct, leading to characteristic T → C transitions that are detected to define protein binding sites [46].

A number of methods have been specifically developed to resolve crosslink events at the single nucleotide level. Individual nucleotide resolution CLIP (iCLIP) and variations thereof make use of the propensity of less processive reverse transcriptases to terminate at (=immediately before) the crosslinked nucleotide [34, 38, 47–49]. RNA hybrid and individual-nucleotide resolution UV crosslinking and immunoprecipitation (hiCLIP) [36] represents an iCLIP-based protocol that has been designed specifically for identifying RNA duplexes bound to RBP(s). Individual nucleotide resolution crosslinking and affinity purification (iCLAP) [32] closely mirrors the iCLIP protocol with the exception of using protein tags for a two-step affinity purification under denaturing conditions. Similarly, crosslinking and analysis of cDNAs (CRAC) [31] as well as ultraviolet crosslinking and affinity purification (uvCLAP) protocols [50] also use protein tags with denaturing affinity purification. Protocols such as Infrared CLIP (irCLIP) [51] and bromodeoxyuridine CLIP (BrdU-CLIP) [52] were established to improve the crosslinking efficiency and library construction steps. PAPER-CLIP adopts sequencing library construction steps from BrdU-CLIP and sample preparation, immunoprecipitation, SDS–PAGE and RNA extraction steps from HITS-CLIP along with modifications implemented to improve the sensitivity [33].

A major advancement introduced by the enhanced CLIP (eCLIP) [40] protocol was the inclusion of a “size-matched input control” (SMInput or SMI), a library generated from a small aliquot of the pre-immunoprecipitation sample run on the polyacrylamide gel and recovered from the same mass window as the RNA bound to the RBP of interest. eCLIP omits in-gel visualization of RBP–RNA complexes, a step commonly included in iCLIP (and related protocols), thereby reducing the overall time required to complete the experiment [40] at the expense of losing traceability of the purified material. Analysis of multiple CLIP and iCLIP datasets of canonical RBPs revealed that over 80% of the reads (cDNAs) truncate at crosslink sites [53], while the remainder extend through these sites. An eCLIP variant, monitored enhanced CLIP (meCLIP), was designed to differentiate crosslink-induced truncation events from read-through events [54]. The analysis of libraries generated for eIF4A3 revealed that the percentage of read-through events varied across conditions between 2% and 24%. The conditions influencing this variability include the type of reverse transcriptase used, and library preparation: whether done by two different experimenters or by the same experimenter at different time points [54]. Additionally, the proportion of read-through events varied also between replicates [54]. iCLIP2 [34] refines the original iCLIP protocol by incorporating advancements such as the introduction of a polymerase chain reaction (PCR) reaction for cDNA pre-amplification before size selection (from irCLIP), and the adoption of independent adapter ligation and cDNA purification steps (from eCLIP) [34].

Further developments in this field include sCLIP [55], which omits steps such as radioactive RNA IP visualization, RNA ligation and cDNA size selection and uses a linear *in vitro* transcription amplification reaction; and easyCLIP, which allows multiplexing multiple proteins in a single experiment [56]. Based on the premise that the frequency of interactions for an RBP with an RNA should be different to that of a non-RNA binding protein, the easyCLIP protocol utilizes the distribution of crosslinking of average non-RNA binding proteins to distinguish specific RNA targets of an RBP [56]. Tailing and ligation of cDNA molecules CLIP (TLC-CLIP) was established to optimize the enzymatic reaction steps in CLIP protocols, and utilized a bead-based single tube strategy to minimize sample loss before amplification [57]. Kinetic crosslinking and immunoprecipitation (KIN-CLIP) [37] was developed to study the binding kinetics (association and dissociation rates) of RBPs and target RNAs. To achieve this, KIN-CLIP employs a pulsed femtosecond UV laser for fast crosslinking and utilizes time course experiments at various protein concentrations and crosslinking efficiencies [37].

Protocols to identify the subcellular localizations of RNA–RBP interactions have also been developed. Proximity-CLIP [30] is a combination of the PAR-CLIP protocol with APEX2-mediated proximity biotinylation [58] to study the localization of RNA–RBP interactome at the subcellular level. Similarly, co-localization CLIP (coCLIP) [59] also utilizes APEX2 proximity labeling to study the functions of RNA–RBP interactions at the subcellular level. While proximity-CLIP enables the identification of all compartmentalized RNA–RBP interactions, coCLIP is tailored for the identification of RNA–RBP interactions at specific, predetermined subcellular locations [30, 59]. Comprehensive reviews and more detailed comparisons of the major CLIP and related protocols are available [60–62].

The protocols discussed so far were designed to examine single RBPs at a time, whereas a couple of the recently published protocols describe “CLIP”ing of several RBPs in parallel, in the same sample. In antibody barcode eCLIP (ABC), DNA-barcoded antibodies were leveraged to characterize different RBPs within the same sample thus reducing the amount of input material required per RBP, as well as simplifying several steps of the protocol [63]. Split and Pool Identification of RBP targets (SPIDR) utilizes split-pool barcoding along with antibody-bead barcoding to multiplex several RBPs in a single experiment [64]. Split-pool barcoding was developed in single cell genomics to uniquely barcode transcriptomic sequences from thousands of cells in a cost-effective manner, without the need for complex instruments. It involves repeated rounds of randomly distributing cells into wells, followed by the addition of well-specific barcodes and pooling [65].

### Non-crosslinking methods

Although UV crosslinking has become a popular technique to study RNA–protein interactions, alternative protocols to CLIP have also been developed. Targets of RNA-binding proteins identified by editing (TRIBE) relies on the transgenic expression of a fusion protein generated by coupling the RBP of interest with the catalytic domain of an RNA-editing enzyme (ADARcd) [66]. This catalytic domain locally deaminates adenosine to inosine close to the RBP binding sites on RNAs, and the specificity of this editing is primarily governed by the RNA recognition features of the RBP. Standard transcriptomic RNA-seq then identifies RNA edit sites enriched for the signature A → G mutations [66]. In HyperTRIBE, a modified version of the TRIBE protocol, the catalytic domain (ADARcd) carries the hyperactive E488Q mutation, which has been shown to increase the number of detected editing sites by up to 20-fold in comparison to TRIBE [67]. Surveying Targets by APOBEC-Mediated Profiling (STAMP) is yet another crosslinking-independent method making use of fusing RNA editing domains to RBPs of interest [68]. The major difference between TRIBE and STAMP protocols is the nature of the RNA editing enzyme: in STAMP, APOBEC1 is employed, which catalyzes cytosine to uracil (C → U) conversions on single stranded RNA substrates and has been used to identify RNA–RBP interactions in single cells [68]. A related development in this field is the TRIBE–STAMP [69] protocol, which can concurrently identify mRNAs binding to two different RBPs by utilizing the unique mutation signatures from both TRIBE and STAMP, respectively. In contrast to these protocols, MAPIT-seq relies on an antibody-directed RNA-editing strategy for the *in situ* investigation of endogenous RBP targets [70]. For this purpose, a recombinant protein fusing both human ADAR2 deaminase domain (with the hyperactive E488Q substitution) (hADAR2dd) and rat APOBEC1 (rAPOBEC1) with protein A/G (pAG) is utilized [70].

### Puzzling the pieces together: CLIP data analysis

#### Data processing and statistical analysis

**Figure 3.**
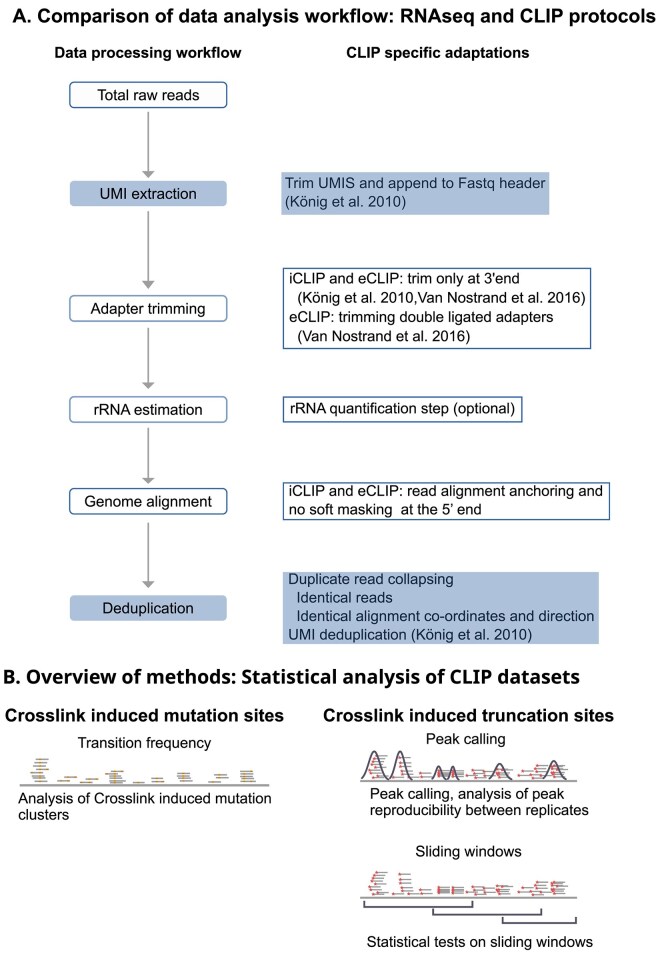
A simplified overview of CLIP data analysis. (**A**) Comparison of RNA-seq and CLIP data analysis workflows. The first column outlines typical RNA-seq steps (rectangles with blue outline) alongside CLIP-specific steps (rectangles with a blue fill). The second column provides additional details and references for CLIP-specific modifications at each step. (**B**) Overview of the statistical analysis methods available for CLIP datasets. For crosslink-induced mutation sites (CIMS), analysis typically involves comparing mutation sites between crosslinked and non-crosslinked control samples. For crosslink-induced truncation sites (CITS), peak calling is commonly used, although additional custom tools employing window-based approaches have also been developed.

CLIP data analysis workflows have heavily adopted strategies from other well established fields such as RNA-seq. Here, we give a brief overview of some of the widely used software suites and standard data analysis practices in the CLIP field. Compared to conventional RNA-seq data processing workflows, one of the striking differences of some CLIP workflows is the handling of Unique Molecular Identifiers (UMIs) (Fig. 3A). UMIs are random sequence barcodes ligated to the reverse transcripts before PCR amplification steps, and are used to identify PCR duplicates during the data analysis steps [38]. Tools such as UMI-Tools [71], Je [72], and bctools (https://github.com/dmaticzka/bctools) are designed for UMI removal and UMI-based deduplication after genome alignment (Fig. 3A). Steps such as adapter removal and read quality trimming can be performed using well known software packages such as Cutadapt [73], Trimmomatic [74], Trim Galore (https://github.com/FelixKrueger/TrimGalore), or fastp [75], and the quality of the reads both pre- and post-filtering can be assessed using a combination of FastQC (https://www.bioinformatics.babraham.ac.uk/projects/fastqc/) and MultiQC [76]. Data analysis pipelines for the comprehensive processing of CLIP datasets, starting from quality control of raw reads to the final statistical analysis, have also been developed. Notable examples of these are FAST-iCLIP [77], Skipper [78], racoon_clip [79], and nf-core/clipseq [80].

For RNA-seq data analysis, bad quality bases at both 5′ and 3′ end of the reads are trimmed off. But, during read quality trimming for iCLIP/eCLIP derived datasets, crosslink-induced truncation sites at the 5′ end of the reads have to be taken into account and, hence, a general recommendation is to trim low quality bases only at the 3′ end of the reads (Fig. 3A). After quality trimming, FAST-iCLIP [77] and Skipper [78] pipelines map reads to repeat region sequences and trim off reads that map to these regions, whereas, racoon_clip [79] maps the trimmed reads directly to the genome. The reads remaining after these steps could be mapped either to the transcriptome or the genome. A major advantage of aligning to transcriptome over genome is the sensitivity of the alignment, with the caveat that only annotated mature transcripts are considered for alignment. However, alignment to the genome preserves binding sites in introns (pre-mRNA transcripts) and un-annotated regions of the genome. Hence, the recommendation is to typically align reads to the genome instead of the transcriptome (with sufficient sensitivity) [81]. Additionally, genome alignment can circumvent issues caused by different transcriptome annotations and also makes visualization by IGV or similar genome browsers easier [82]. Reads that span splice junctions can be mapped to the reference genome using splice-aware aligners such as STAR [83] or HISAT [84]. For iCLIP/eCLIP datasets, the aligner parameters have to be adjusted so that the 5′ end of the reads is not masked from alignment (since crosslink site is assumed to be one base upstream of the 5′ end), but is always mapped to the reference genome (Fig. 3A).

Genome alignment is followed by the identification of target RNA regions binding to the RBP. Analogous to ChIP-seq data analysis, the CLIP field has also adopted the term “peak calling” to denote the identification of RNA regions with significantly higher than expected crosslink events over the background or control dataset. Detailed reviews of major peak calling algorithms are available [62, 81, 82]. Statistical analysis packages designed for PAR-CLIP data quantify the T → C conversion rates in aligned reads. Notable algorithms developed for the analysis of PAR-CLIP data include PARalyzer [85], CIMS analysis [43], and wavClusteR [86]. Peak callers such as CLAM [87], Clippy (https://github.com/ulelab/clippy), CLIP Tool Kit [88], PIPE-CLIP [89], Piranha [90], pyCRAC [91], and omniCLIP [92] were designed to handle data from multiple CLIP protocols, whereas, peak callers such as CLIPper [93], iCount [32], PureCLIP [94], RCRUNCH [95], and Skipper [78] were designed primarily to analyze peaks from either iCLIP or eCLIP experiments. The majority of these analysis packages can handle only a single replicate at a time, however, packages specifically designed for the quantitative comparison of CLIP data with replicates have also been developed. dCLIP [96], a two-step bin (window)-based analysis model divides read clusters into bins of small length (5 bp), and uses a modified MA-plot approach for data normalization. dCLIP then applies a hidden Markov model to detect RBP-binding regions that are either shared across conditions or specific to a single condition [96]. Recently, we introduced a sliding window-based approach for CLIP region enrichment called DEWSeq [97], where we split annotated regions of the genome into overlapping windows of equal length and summarize the number of crosslink sites in these windows. We adapted the DESeq2 [98] package for library size normalization and statistical testing across different conditions. An analysis approach that combines peak calling with statistical testing across multiple samples has also been developed. BindingSiteFinder (https://github.com/ZarnackGroup/BindingSiteFinder) calls consensus peaks on merged datasets using a peak caller such as PureCLIP, and computes the differential enrichment for these peaks across IP and control samples using a DESeq2 [98] based statistical approach. Figure 3B summarizes statistical analysis methods available for CLIP datasets.

#### CLIP: use of background controls

The choice of background controls is a major determinant of the outcome identification of enriched peaks/binding regions in CLIP protocols. Possible experimental controls include non-crosslinked samples, non-immunoprecipitated samples, lysates from RBP knockout samples, IPs after mock treatments, or IPs using isotype-matched IgG. Depending on experimental conditions, some may be difficult to obtain or only moderately informative [46, 99]. In the original HITS-CLIP protocol, the RNA tags that remained in supernatants after Nova immunoprecipitation (which corresponds to a sample of all RBP–RNA interactions in the brain) were used as a control to compare the frequency of Nova binding sites for HITS-CLIP data [41]. RNA-seq analysis of the corresponding cell line or tissue to account for background T→C mutations has been proposed as a control for PAR-CLIP data [46].

For iCLIP data analysis, background controls are generated by randomizing crosslinked nucleotide positions *in silico*, taking into account transcript abundance. To account for the inherent difference in expression and crosslink site distribution among various gene features, randomizations are done within each gene feature separately, ensuring that crosslink sites in exons are separated from those in introns, and those in untranslated regions from the sites in coding sequences [32, 38]. Similarly, irCLIP and BrdU-CLIP also employ *in silico* randomized crosslink sites as background controls for the identification of crosslink events [51, 52]. iCLIP assesses reproducibility by comparing the similarity of crosslink counts at a given site/nucleotide across two or three replicates [32, 38]. eCLIP and associated methods use SMIs as key controls for nonspecific background noise as well as several other intrinsic biases, and irreproducible discovery rate (IDR) [100] analysis is used to measure the reproducibility across biological samples [40]. The benefits of SMI as a background control were demonstrated by analysis of the binding sites of the stem loop binding protein (SLBP), where only 1.2% (284) out of the total identified peaks in the datasets (23 034) were enriched over the SMI control [40], indicating SLBP-independent enrichment in IP samples. In TLC-CLIP, peaks from libraries generated omitting the ligation step, IgG controls as well as ENCODE blacklisted regions from eCLIP libraries, were used to filter out nonspecific enriched regions [57].

Since gel and membrane transfer steps, fundamental to generating conventional SMI control libraries in eCLIP, are absent from the ABC protocol, controlling for nonspecific binding using SMIs is not possible. To identify specific binding sites for a single RBP in ABC, the binding site information from all other multiplexed RBPs is being used as an alternative control, referred to as “complement control” [63]. A similar strategy with random downsampling was adopted in SPIDR to control for nonspecific binding of an RBP [64].

#### Discovering the binding context

The identification of differential binding regions/enriched peaks is followed by the identification of the element that directly interacts with the RBP of interest. At least for most classical RBPs, this element represents an RNA sequence and/or structure motif, while several non-canonical RBPs have recently been found to specifically bind RNA sequences lacking identified common denominators [101]. In a study analyzing the target preferences of 78 canonical human RBPs, the following binding characteristics were revealed: (i) in general, RBPs preferentially bind to sequences with low compositional complexity, (ii) RNA sequence context impacts on RBP binding, (iii) many (canonical) RBPs interact with short spaced motifs (bipartite motifs), (iv) RBPs exhibit a wide range (modest to extreme) of sensitivity to RNA structures, and (v) RNA structural elements (hairpin loops, stems, bulged stems) influence the binding of some RBPs [102]. General *de novo* motif discovery and analysis suites such as the MEME Suite [103] and HOMER [104] have already been used to predict and analyze RBP binding motifs. The dynamic nature of RNA secondary structures makes them more challenging to analyze and interpret from CLIP results. Nevertheless, a selection of software packages are available for the analysis of secondary structures from CLIP results [105–107]. Several deep learning models were also introduced in the past few years to predict the binding preferences of RBPs [108–110]. Major computational tools available for the analysis of RNA–protein interactions as well as RNA–protein interaction databases have already been reviewed [111, 112]. However, the *in silico* discovery of motifs or the prediction of secondary structures represent only initial steps in identifying the binding and functional elements of RNA. To gain a comprehensive understanding of RBP binding and its functional context, integrative data analysis approaches, such as those employed by [113], along with complementary wet lab experiments are essential.

It is important to consider that while most of the computational post-processing steps are geared toward identifying the common features of targets, such as linear motifs or secondary structures, non-canonical RBPs appear to bind RNA without direct sequence specificity. For example, the glycolytic enzyme ENO1 interacts specifically with a collection of mRNAs without a common sequence motif [15]. This result is in accordance with a large-scale analysis of the binding preferences of 492 human non-canonical RBPs, where it was shown that only 23 (4.7%) RBPs showed sequence specificity in binding [114]. To shed further light on this matter, the binding profiles (clear peaks versus broad binding) of such non-canonical RBPs need to be studied in detail. Additionally, the impact of mechanisms such as co-transcriptional loading could also be explored.

## Bumping into hurdles: challenges in CLIP field

While CLIP methods facilitate the identification of direct targets of an RBP, it is also equally important to recognize their limitations and challenges, which are outlined in this section. A summary of the major discussion points in this section is available in Table 1.

**Table 1.** A brief overview of major challenges in the CLIP field described in this manuscript

Major challenges in CLIP field	Condensed description
**Transient versus stable interactions**	A multitude of factors determine the dynamic nature of protein–RNA interactions [115]
	No clear distinction between transient and stable interactions [62]
**UV crosslinking**	Crosslinking inefficiency (only 1%–5% of protein–RNA contact points) [116]
	High input material requirements compared to chemical crosslinking [117]
	Tendency to stabilize transient protein–RNA interactions; associated false-positive signals (examples: MALAT1, XIST) [118]
	Difference in crosslinking efficiency among various RBPs [119, 120]
	Bias toward crosslinking Uridine-rich regions; poor crosslinking of double stranded RNAs [53, 121, 122]
	Occasional protein–protein crosslinking [123, 124]
**RNA digestion**	Overdigestion or RNA fragments leading to poor mapping and underdigestion of RNA fragments leading to poor library preparation [60, 125]
**Immunoprecipitation (IP)**	Lack of proper validation of commercially available antibodies for IP [82]
	Limited antibody specificity [60] and inefficient IP [99]
	Supraphysiological expression of the epitope-tagged protein and potential interference of the tag with RNA-binding activity, subcellular localization, or condensation properties [117, 126]
**Library preparation**	Inefficient adapter ligation requires many additional rounds of amplification [99] leading to PCR amplification biases in the final library
	Low predictability of crosslink site truncation versus read-through events (iCLIP, eCLIP) [54]
**Data analysis challenges**	
**Data pre-processing**	PCR duplication events limiting the number of mapped reads for statistical analysis
	Less than 50% of total input reads retained in downstream analysis (Fig. 4)
	eCLIP: two step adapter trimming due to intermittent adapter double ligation events [40], leading to loss of reads
	Contamination from external sources in low RNA yield samples [127]
**Statistical testing and background selection**	IgG and no-crosslink controls yield negligible RNA amounts for library preparation [99] and are therefore not meaningful background controls
	Drawbacks of non-specific controls (RNA-seq) compared to specific background (input control or SMI): non-specific binding cannot be filtered out using RNA-seq as a background control [82]
	Possible bias of SMIs toward RBP binding preferences [128]
**Result reproducibility**	Low reproducibility of peaks/regions between replicates, CLIP methods, and between CLIP and non-crosslink methods [97]
	Discrepancy among peak calling, statistical testing algorithms on the same dataset [82]
**Binding context**	RBP specific differences between crosslink site and binding region [97]
	Presence of CL-motifs (Uridine-rich motifs occurring as an artefact of UV crosslinking) [125]
	Potential noise from co-binding RBPs
	Challenges in incorporating RNA structural context [81] and post-transcriptional modifications [129] to binding preferences
	Binding without sequence specificity [15, 114]

### Transient versus stable interactions

Numerous parameters such as the respective concentrations of the interaction partners, the affinity of the RBP for the RNA binding element, competition (if present) from other RBPs for the RNA binding site and, conversely, competition from other RNAs for binding to the RBP, determine the dynamic nature of interactions between RBPs and RNAs [115]. Kinetic parameters describe the dynamics of protein–RNA interactions at the molecular level. The association and dissociation rate constants for protein–RNA complexes can vary dramatically [130, 131], resulting in interactions that range from very transient to highly stable. However, the boundaries between these categories are not clearly defined [62], making the differentiation of a stable interaction from a transient one challenging. Furthermore, crosslinking converts a transient interaction into a stable (covalent) one, aggravating this challenge.

### Crosslinking-associated challenges

UV-crosslinking creates covalent bonds between amino acids and nucleotides in very close proximity (“zero length”) within microseconds [132, 133]. Although this property facilitates the isolation of RBP-bound RNAs under stringent conditions, UV-crosslinking is inefficient (crosslinking only 1%–5% of protein–RNA contact points) [116]. Additionally, the amino acid-nucleotide interactions underlying this zero length crosslinking are still not fully understood. Major challenges include: (i) the ongoing difficulty in the simultaneous identification of the precise crosslink sites on both the protein and the RNA and (ii) the inefficiency and lack of predictability associated with RNA–RBP UV-crosslinking [134, 135]. While crosslinking followed by mass spectrometry methods exist for the simultaneous identification of the crosslink sites on both the protein and the RNA, these techniques face technical challenges that limit their efficiency and coverage [135, 136], or they are limited to the analysis of purified individual RNA–protein complexes [134]. To boost the UV-crosslinking efficiency and signal strength, certain CLIP protocols use high UV-C crosslinking energies for cells in a monolayer. However, this approach increases the risk of multiple RBPs crosslinking to the same RNA, leading to the co-purification of contaminant RBPs [60]. Such inadvertent co-purification of contaminant RBPs could lead to the identification of false-positive motifs as binding targets, bound by the contaminant RBPs, or to the detection of false crosslink sites. The use of photoactivatable ribonucleosides (4SU or 6SG) in PAR-CLIP restricts the applicability of this protocol to biological systems where these nucleosides can be efficiently incorporated. Additionally, the proximity of these modified bases to the RNA binding site on the RBP can affect crosslinking [60]. An interactome capture study comparing conventional UV-crosslinking (254 nm) with 4SU-mediated UV-crosslinking found that out of 1316 RBPs identified in total, 64% were identified by both crosslinking methods, 24% (316 RBPs) were found exclusively by conventional crosslinking, while 12% (158 RBPs) were unique to the PAR-crosslinking datasets [137]. These findings suggest that relevant differences in the propensity of RBPs exist toward the crosslinking techniques used, and therefore, further investigation is required to assess the impact of these differences on CLIP-seq protocols. When compared to chemical (formaldehyde) crosslinking, UV crosslinking is less likely to capture protein–RNA interactions, hence, more input material (cells) might be required to capture these interactions [117]. Although UV irradiation is predominantly thought to crosslink proteins to nucleic acids, studies suggest that UV light can occasionally also induce protein–protein crosslinks [123, 124], potentially leading to the co-purification of protein contaminants and associated RNAs. As discussed in the previous section, another caveat associated with crosslinking is the potential to covalently stabilize transient protein–RNA interactions that lack biological significance. Detected signals (reads) corresponding to transient, nonspecific interactions with highly abundant RNAs can, in fact, overshadow the signal associated with genuine RNA targets that are far less abundant. For example, the abundant long non-coding RNAs MALAT1 and XIST were identified as targets in several PAR-CLIP and iCLIP studies [118]. For this reason, it is desirable to validate RNA targets identified by CLIP using a crosslinking-free approach, such as RNA immunoprecipitation followed by RT-qPCR.

The UV-crosslinking efficiencies of RBPs vary dramatically. For instance, when compared to the respective input controls, HuR can be efficiently crosslinked (up to 1% of the cellular protein can be crosslinked to RNA), whereas hnRNPL crosslinking is less efficient (only ~0.1% of the cellular protein is crosslinked) [119]. A recent study introduced a metric called protein-specific UV-crosslinking efficiency (%CL), defined as the ratio of RNA-bound protein abundance to the total protein abundance, to quantify the molecular fraction of a protein that is crosslinked to RNA [120]. Analysis of 1097 proteins in this study revealed that %CL values can show up to five orders of magnitude variation, ranging from %CL value of 45.9 for HuR to 0.0016 for the metabolic enzyme GAPDH [120]. A notable discrepancy between the two aforementioned studies is the crosslinking efficiency reported for HuR (1% versus 45.9%). This difference could stem from the purification and quantification methods used in each case. The first study estimated the recovery of crosslinked RNP complexes to be around 25%–30% and used western blotting based quantification to compute the relative enrichment [119]. In contrast, the second study reports a near 100% recovery of the total protein, RNA and the crosslinked RNP complex, and employed quantitative mass spectrometry for relative enrichment analysis [120]. Despite these methodological differences, both studies underscore the substantial heterogeneity in crosslinking efficiencies among RBPs.

The marked difference in crosslinking efficiency across RBPs is attributable to the amino acid and nucleotide composition of the protein–RNA interfaces. While nearly all amino acids (except for aspartic acid, asparagine, glutamic acid and glutamine) have been found to crosslink to nucleotides [121], they do so with different efficiencies [136]. Conversely, uridine represents the predominantly crosslinked nucleotide [53, 121], skewing RNA molecules retained after crosslinking in favor of U-rich regions. Additionally, double-stranded RNA is poorly UV-crosslinked compared to single-stranded RNAs, due to the non-availability of the hydrogen-bonded nucleotide bases [122]. Moreover, the protocol (CLIP vs iCLIP) used can strongly influence the nucleotide enriched at the crosslink site [53]. Accurate determination of crosslink-induced truncation sites at the 5′ end of the reads forms the basis for the analysis of data from iCLIP, eCLIP and related protocols. However, analysis of reverse transcriptase read-through events at the crosslink sites in meCLIP libraries showed that the frequency of such read-through events cannot be predicted even for a single RBP [54]. Consequently, in iCLIP/eCLIP datasets, high frequencies of such read-through events can compromise the precise identification of crosslink sites, thus affecting the reliability of binding site analysis. In addition to crosslinking efficiency, the abundance of the RBP in question is yet another factor influencing the complexity of RNA–RBP interactions. Highly abundant RBPs are more prone to nonspecific crosslinking events, whereas RBPs with low crosslinking efficiency are more susceptible to crosslinking events from co-purified RBPs.

In CLIP protocols, UV-crosslinking is followed by cell lysis and RNA digestion steps. The choice of RNases used in the digestion step varies among different protocols, and the sequence specificity of the enzymes can bias the assignment of binding sites [138]. Overdigestion with RNases affects RNA lengths, narrowing the distribution of cDNA sizes in the final library and potentially compromising read mapping. Analysis of an eIF4A3 iCLIP suggests that when the cDNA fragment size distribution is constrained, the resulting crosslink sites may not fully span the binding site of the RBP, leading to a narrow assignment of the binding regions [125]. On the other hand, underdigestion leaves longer RNA fragments that fail to properly amplify during library preparation [60].

For CLIP analyses of endogenous RBPs, high quality of the antibody used is essential. According to Chen *et al*., a significant limitation of CLIP analyses is the availability of high-quality antibodies for the RBP of interest, as many commercially available antibodies are not thoroughly validated for immunoprecipitation [82]. Therefore, the effectiveness and specificity of these antibodies as well as the buffer conditions used for the immunoprecipitation need to be validated in preliminary experiments [82]. Specificity can be compromised not only by high levels of contaminant RBPs, but even by low levels of contaminant RBPs with significantly higher crosslinking efficiencies than the RBP of interest [60]. While antibody quality and specificity issues can be tackled by the use of epitope tags, the physiological expression of the tagged protein and the possibility that RNA binding is affected by the tag have to be taken into account [117, 126]. The low efficiency of immunoprecipitation and subsequent adapter ligation steps often require many rounds of amplification of CLIP libraries to generate the amount of material (cDNA) required for sequencing [99], which may introduce PCR amplification biases in the final samples. Because only experiments with positive outcomes are reported in the literature, an estimation of the failure rate of different CLIP protocols is hard.

### Data analysis challenges

Next, we wish to discuss some of the major challenges associated with the processing and analysis of data from high-throughput CLIP experiments.

#### Data pre-processing

As discussed above, the analysis of CLIP-seq data is challenging. In principle, any RNA can interact with any protein to some extent, and the high abundance of either the RNA or the protein could result in the detection of low-affinity interactions without biological function [117]. A crucial step in data analysis is to determine the quality of the sequencing data. Generally, RNA-seq datasets employ a single round of trimming to remove adapter sequences and low-quality bases at the end of the reads. In eCLIP data, intermittent adapter double ligation events and the ligation of multiple 5′-truncated barcode fragments at the 5′ end of the Read 1 are observed ([40] Supplementary protocol S2). These technical artifacts typically necessitate an additional adapter trimming step that includes various barcode truncations to remove the problematic sequences ([40] Supplementary protocol S2). In our experience with eCLIP (and seCLIP) data, 20%–30% (or more) of the total input reads are typically removed during this two-step adapter trimming. The impact of these issues on data quality and the data loss introduced needs to be carefully evaluated. As mentioned in the previous section, pipelines such as FAST-iCLIP [77] and Skipper [78] trim-off reads mapping to repeat regions for further analysis, whereas racoon_clip [79] pipeline omits this step. Along these lines, an analysis performed on 223 eCLIP datasets showed that the percentage of reads aligning to repeat regions spans a wide spectrum: from 10.4% of reads (for KHSRP) to 95.2% (for RPS11) [139]. Given this variability, it is challenging to assess the general applicability of excluding reads mapping to repeat regions, and this decision must be taken on a case-by-case basis.

**Figure 4.**
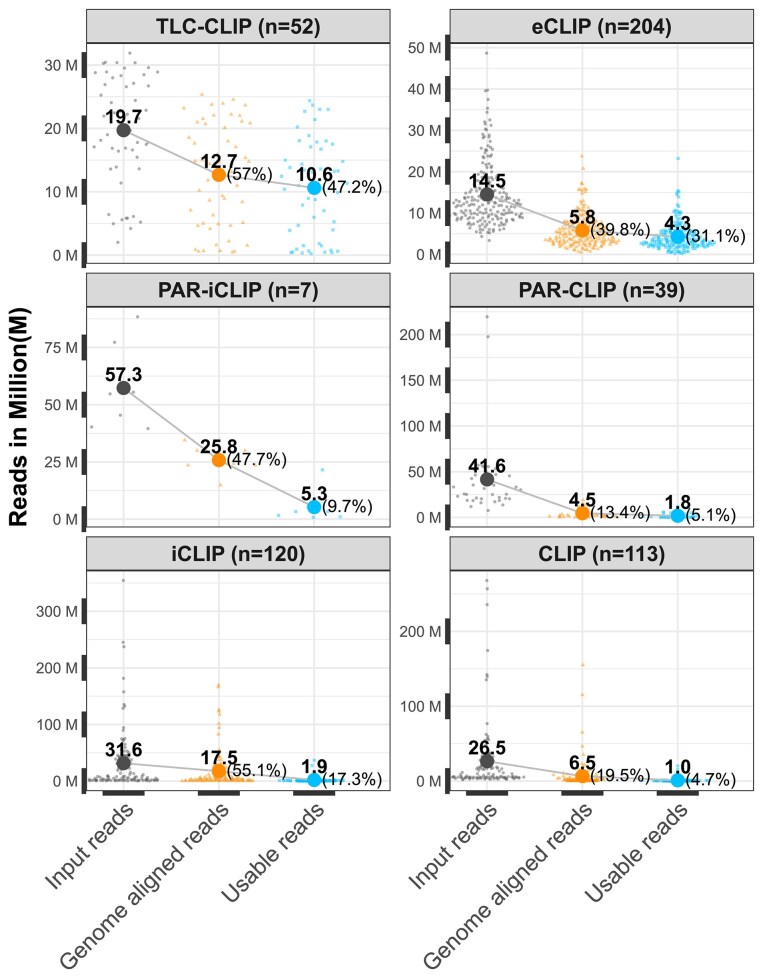
Comparison of reads retained across different CLIP protocols. Our analysis focused on these protocols based on data availability. Note that the number of datasets available for each protocol were different, with the number of studies available per protocol indicated in the panel facets. Each panel presents the read counts at three key stages of the data analysis pipeline for each protocol: Input reads (total raw reads, represented by black dots), genome-aligned reads (golden dots), and usable reads (uniquely mapped and deduplicated, shown as blue dots). Mean read counts at each step are represented by large dots in corresponding colors and are labeled with their respective values. For genome-aligned reads and usable reads, the percentage of mean read counts relative to the total reads is given in parentheses. The proportion of genome-aligned reads relative to total input reads exhibits a protocol-specific trend. PAR-CLIP datasets have the lowest mapping rates: from an average of 41.6 million total reads, only 4.5 million (13.4%) map to the genome, out of which only 1.8 million (5.1% of total) are usable. In eCLIP, ~5.8 of 14.5 million reads (39.8%) map to the genome, with 4.3 million usable reads (31.1% of total). Standard CLIP datasets show an average of 26.5 million total reads, with 6.56 million (19.5%) mapping and 1 million usable (4.7%). In the TLC-CLIP dataset, 12.7 of 19.7 million reads (64.5%) map to the genome, and 10.6 million (47.2%) are usable. PAR-iCLIP datasets contain 57.3 million total reads on average, with 25.8 million (47.7%) mapping and 5.7 million (9.7%) usable. Finally, iCLIP datasets average 31.6 million total reads, with 17.5 million (55.1%) mapping and 1.9 million usable (17.3%). Note that the read statistics for CLIP, PAR-CLIP, PAR-iCLIP, iCLIP, and eCLIP protocols were sourced from Supplementary Tables S1 and S2 in [40], and those for TLC-CLIP from Supplementary Table S2 in [57]. Consequently, they do not include data published since 2016 and 2023, respectively. More recent data can be sourced from the Flow.bio platform (https://app.flow.bio/), which provides an extensive collection of current publicly available CLIP-seq datasets, along with the associated statistics. The source code and the list of data sets used to generate this figure are available in the following github repository: https://github.com/tsekara/CLIP_metaanalysis.

Another major challenge in CLIP data analysis is the bias introduced by the PCR duplicates. These artifacts can significantly limit the number of uniquely mapped reads and hence, informative sequences available for statistical analysis after UMI-based deduplication. In general, optimizing PCR cycle numbers and the use of UMIs can help mitigate such artifacts [62]. To get an overview of the number of reads retained at critical steps in the data processing workflows, we compared the available read mapping values for various CLIP protocols (Fig. 4). For this analysis, read statistics for CLIP, PAR-CLIP, PAR-iCLIP, iCLIP, and eCLIP protocols were retrieved from Supplementary Tables S1 and S2 in [40] and for TLC-CLIP from Supplementary Table S2 in [57]. Our analysis revealed significant data loss during processing across all protocols. Within CLIP, PAR-CLIP, and PAR-iCLIP datasets, the average number of usable reads (uniquely mapped and deduplicated reads) was <10% of the total input reads: 1, 1.8, and 5.3 million, respectively. In the iCLIP datasets, the average number of usable reads was 1.9 million, corresponding to ~17.3% of the input reads. The eCLIP dataset had an average of 4.3 million usable reads, accounting for ~31.1% of the input reads. The TLC-CLIP datasets exhibited the highest number of usable reads, with a mean of 10.6 million, retaining an average of 47.2% of the input reads.

It is important to realise that the direct comparison of these technologies on a broader scale is limited by the lack of direct side-by-side comparison studies. Therefore, we recommend not to draw comparative conclusions. However, the numbers clearly indicate that, across all evaluated protocols, <50% of the total input reads are commonly retained for downstream analyses. The read statistics shown in Fig. 4 are based on a metaanalysis of published data [40, 57]. The Flow.bio (https://app.flow.bio/) platform hosts a collection of current publicly available CLIP-seq datasets, along with read statistics and associated results. A controlled study including a sufficient set of canonical and non-canonical RBPs comparing multiple well-established protocols as the only variable would be valuable to gain a deeper understanding of protocol benefits and limitations on the final outcomes.

In CLIP datasets derived from sources with low RNA yield, such as RBPs with low crosslink efficiency or samples/cell types that inherently yield low amounts of RNA, contamination from external RNA sources also needs to be considered. One example of such a contamination was reported by [127], where sequences from *Acinetobacter johnsonii* XBB1 (CP010350.1) were identified as the predominant contaminant in CLIP experiments from (low RNA yield) human embryonic stem cells. The source of this contamination was traced back to the nitrocellulose membranes used in the protocol [127]. Consistent with this, a pilot study conducted in our lab (unpublished data) using nitrocellulose membranes also detected *Acinetobacter johnsonii* XBB1 (CP010350.1) as the major contaminant.

#### Statistical testing and background selection issues

Background/noise controls should be selected to remove random crosslink events generated by the mechanisms discussed above (refer to the “transient vs stable interactions” and “Crosslinking-associated challenges” sections). ChIP-seq experiments for DNA-binding proteins have often used an IgG as a background/negative control. The use of IgG controls in CLIP-seq experiments has however proven to be of limited value, as the IP in the absence of a pulled-down protein yielded negligible amounts of RNA for library generation [99]. “No crosslink” controls omitting UV irradiation have a similar drawback. Normalization of CLIP data to mRNA-seq data (as a control for the abundance of certain RNAs) can improve the specificity of mRNA binding site assignments for an RBP of interest [140]. However, differences in transcript-level estimations resulting from steps such as poly-A selection or rRNA depletion before RNA-seq require consideration [62]. Compared to a CLIP-specific background (such as SMI or input control), using RNA-seq data (derived from the same sample) as a background control has the additional drawback that the nonspecific binding sites cannot be filtered out [82], as RNA-seq data lack information about crosslinking events. While the original CLIP protocol proposes to use a total input control generated from the RNAs bound to the total pool of RBPs in the cell [141], studies using eCLIP or associated protocols make use of SMI as a background control [40]. However, we have not yet come across published studies that directly compare the pros and cons of using total input control over SMI or vice versa.

Chen *et al*. used Zfp36 eCLIP datasets to evaluate the specificity of the reported binding sites by computing the proportion of the reported regions overlapping a known motif (UAUUUAUU) [82]. This study found that normalizing the regions with respect to SMI greatly enhanced the fraction of binding regions overlapping the known motif for four different peak calling algorithms (CITS, CLIPper, iCount, and Piranha) [82]. However, a potential caveat of using SMI as the control is that for abundant RBPs, the background-noise estimation can be inaccurate if the sequences in SMI samples are dominated by the true binding partners of the RBP of interest [62]. This limitation is highlighted by a study based on positional motifs, which suggested that the effectiveness of SMI as a control may depend on the specific binding preferences of the RBP being investigated. When compared to using non-significant peaks from the same IP sample as a background control, the use of SMI as a control for peak calling resulted in better motif enrichment for RBPs binding to A-rich motifs [128]. In contrast, RBPs that bind to U-rich motifs showed the opposite trend [128]. The use of complement controls as described in ABC and SPIDR is limited to studies that multiplex a sufficiently large number of RBPs, therefore, the applicability of these controls in experiments profiling one or a few RBPs is limited. Finally, the ongoing discourse regarding the RNA-binding activity of PRC2 and the question of how different analysis and normalization approaches can yield contradictory results also warrants further attention [142, 143].

#### Reproducibility puzzles

A real issue in CLIP data analysis is the reproducibility of the results. Based on the analysis of eCLIP binding sites of 150 RBPs published by the ENCODE consortium, we previously reported the relatively low reproducibility of binding sites between replicates [97]. The median percentage of overlapping peaks in both replicates is only around 59%. Within the same study, we compared motif-containing binding sites derived from ENCODE eCLIP data with those obtained from iCLIP, HITS-CLIP, or PAR-CLIP experiments, retrieved from the POSTAR2 database. Our analysis found that the overlap between binding sites from different cell lines and protocols, but pertaining to the same RBP, was low, comprising only 2.1% of the sites [97]. The choice of statistical analysis tools used to determine the binding regions contributes to this problem. A comparison of the top 1500 peaks identified by the peak callers Piranha, iCount, CITS and CLIPper on a Zfp36 eCLIP dataset revealed varying degrees of overlap among the enriched peaks [82]. Similar findings were also reported when we compared eCLIP peaks obtained from CLIPper and DEWSeq analysis packages, iCLIP peaks generated using Piranha and CIMS tools, as well as PAR-CLIP peaks generated using Piranha, PARalyzer, and Mukherjee’s method [97]. Furthermore, when we compared eCLIP regions for TARDBP with results from the non-crosslink methods HyperTRIBE and STAMP, the correspondence between eCLIP-enriched regions and RNA edit sites from the non-crosslink methods were found to be minimal [97].

#### Hurdles in discovering binding context

Several experimental and RBP-specific factors can confound the discovery of RNA elements relevant for RBP binding. It is very important to realise, but not widely appreciated, that the binding site may not directly correspond to the crosslinked region and actually lie either upstream or downstream of it. For RBFOX2, the motif UGCAUG perfectly overlaps most crosslink positions, whereas the crosslink sites for TARDBP are mostly located upstream of its GAAUG binding motif [40]. Our previously published analysis of the 107 RBPs with known binding motifs in the ENCODE eCLIP dataset corroborates this point: although crosslink sites correspond to the known binding motifs for most analyzed RBPs, they can also be located either upstream or downstream of the actual binding sites [97]. Although the reason for this has not been rigorously determined, it is plausible that crosslinks could systematically enrich a near-by region with a more favourable crosslinking chemistry or geometry than the actual binding region. Consequently, motif analysis faces a further challenge, especially for RBPs without known binding sites, due to the risk of overlooking the sites located outside of the immediate crosslink regions.

The analysis of PTBP1 eCLIP mock input data revealed the presence of CL-motifs (UV crosslink-associated motifs) rich in Uridines [125]. These CL-motifs can make the deconvolution of functional motifs of an RBP challenging, particularly when the RBP in question also shows a preference toward Uridine-rich binding motifs. Furthermore, as reported by [35], multiple RBPs can co-bind to the same RNA as the RBP of interest. In such cases, the potential noise introduced by co-binding RBPs in binding site analysis needs to be taken into account. A well-known, yet less understood factor governing RBP binding is the structural context, and it is a daunting task to incorporate the RNA structural complexity and/or the interdependence between structure and sequence into analysis models [81]. Likewise, post-transcriptional modifications on target RNAs can either promote or hinder RBP association, as demonstrated, for example, in the case of *N*^6^-methyladenosine (m^6^A) [129]. Recent studies indicate that the binding preferences of non-canonical RBPs often lack sequence specificity [15, 114]. This lack of a recognisable RNA motif together with the presence of false positives (such as CL-motifs) contributes to the challenges of determining the RNA binding sites of novel RBPs. A summary of the main points discussed in this section along with associated references (where available) are provided in Table 1.

## Unveiling the mysteries of RBPs: a future perspective

With so many challenges, where can solutions come from? There are >20 different variants of CLIP and similar protocols that have been published to date (Fig. 1), each representing a possible improvement over the existing protocols, or a modification tailored to address a specific research question. Although certain protocols (iCLIP, PAR-CLIP, eCLIP, and HITS-CLIP) are more widely used than others (Fig. 2), efforts to comprehensively and systematically evaluate the strengths and weaknesses of each protocol as well as to formulate guiding principles or design considerations to select the most appropriate CLIP variant for an experiment have been minimal. Such resources would be invaluable, especially for research groups inexperienced in the field, as this would help them navigate the growing landscape of CLIP protocols and choose the best option to address their specific biological questions. A preliminary development in this direction would be the benchmarking of iCLIP2 and iiCLIP protocols using the same input material [49].

As discussed above, UV crosslinking is both powerful and suffers from inherent problems. To mitigate these, efforts have focused on enhancing crosslinking efficiency, including the application of pulsed ultraviolet lasers [37], and the development of more efficient irradiation devices [144]. Additionally, complementary strategies to optimize RNA–protein complex purification [119] have also been developed. At this juncture, assessing the RBP’s effectiveness for conventional versus 4SU UV-crosslinking, as previously reported [137] can be beneficial. While current CLIP-seq protocols have evolved from the original CLIP method, their inherent complexity remains a consideration. Factors such as substantial input material requirements, limited RNA recovery post-immunoprecipitation, and the multistep workflow contribute to this complexity. Moreover, each stage introduces potential biases that may result in false positives, false negatives, or both. For instance, small RNAs can be preferentially amplified during library preparation due to their limited size, potentially leading to their erroneous characterization as binding targets for an RBP. A typical iCLIP2, irCLIP, or eCLIP experiment is also time-intensive, often requiring up to four days of experimental work [34, 40]. Considering these limitations, future research could focus on developing streamlined versions of these protocols to reduce complexity, bias, and the number of experimental steps, thereby improving the efficiency and at the same time enabling their broader adoption for a wider range of applications and research communities. Although iCLIP, eCLIP, and related methods offer single-nucleotide resolution of crosslinking events, it is pertinent to consider whether this level of resolution is beneficial to answer the experimental questions at hand. From our perspective, improving the accuracy of binding or functional site detection outweighs the advantages of determining the crosslink site at the single nucleotide level, particularly since crosslink sites and functionally relevant binding sites can differ [97].

A shared drawback of several CLIP data analysis methods is the lack of proper background controls in experiments and the use of *in silico* resampled IP data as the background control. Although there is no gold standard background control for CLIP, the use of any control has been shown to improve the specificity of binding regions. However, studies discussed in the previous section also highlight the potential caveats associated with the use of a non-specific control such as RNA-seq [62] and the advantages of using specific controls such as SMI [82]. Therefore, all CLIP experiments should include appropriate negative controls, such as input controls or SMIs. At this juncture, based on the initial evidence reported by [128] that the effectiveness of SMI as a background control depends on the nature of the RBP, it may also be useful to evaluate whether the binding preference of the RBP under study can inform the selection of an appropriate negative control. Isotype-matched IgG and non-crosslinked controls often provide limited information due to the typically low levels of co-precipitated RNA in the absence of a major pulled-down protein. Likewise, using other RBPs as controls may result in the pull-down of different levels of RNA compared to the RBP under investigation. Here, the potential of using IP samples from an RNA binding-deficient mutant of the RBP of interest as a negative control merits consideration. Alternatively, the possibility of performing *in vitro* CLIP experiments using a recombinant version of the RBP, as performed in [145] could also be explored. However, considering the efforts involved in generating functional protein mutants or establishing *in vitro* experiments, these approaches should be evaluated on a case-by-case basis.

Another aspect in need of refinement is the number of biological replicates used for each investigated RBP. A previous survey highlighted that the number of necessary replicates very much depends on the goal of the experiments, experimental variability, the sequencing depth and the complexity of binding patterns of the RBP of interest [146]. The same survey also argued for rigorous and comprehensive studies to understand the effects of replicates on statistical power and accuracy of binding site detection [146]. Although power analysis and sample size estimation methods for RNA-seq [147], ChIP-seq [148], and general recommendations for omics and sequencing data [149] have been published, we are yet to come across publications focused on CLIP-related protocols. Considering the potential value of such work, this issue warrants thorough consideration.

On the data analysis side, a comprehensive evaluation of the statistical approaches used in the field is also necessary. Discourses such as whether the accumulation of crosslink sites into peaks is a generally observable phenomenon in CLIP datasets or whether a comprehensive analysis of binding modes of different RBPs is necessary merits attention. In some CLIP studies, data from replicates are pooled together for binding site detection, a proclivity that was also reported by [146]. Despite their call for advanced statistical methods that make use of the biological replicate information in CLIP data analysis, the tendency to pool replicates for data analysis is still quite prevalent. In our opinion, pooling replicates is not recommended, as it obscures biological variability and can result in the detection of apparently significant binding sites that are, in fact, sample-specific artifacts that are not reproducible across replicates. Furthermore, reporting the reproducibility between peaks from different replicates using methods such IDR analysis [100], as well as a more meticulous review process for CLIP datasets could also be considered. In this context, it is also crucial to evaluate whether the observations made regarding sequencing data in RNA-seq and ChIP-seq are valid for CLIP data, or whether a partially or an entirely different approach would be beneficial to handle CLIP data. As we discussed in the previous section, different tools used for detecting binding regions show varying degrees of consistency when applied to the same data. Method developers often rely on custom datasets or *ad hoc* benchmarking criteria, as the CLIP field lacks gold standard datasets for method comparison and benchmarking. Recent development of motif based analysis and benchmarking strategies such as positionally enriched k-mer analysis (PEKA) [128] and RBPBench (https://backofenlab.github.io/RBPBench/) highlight ongoing efforts to address this issue. A concerted, community-wide effort is required to generate common datasets and standards for CLIP protocols, as well as for the comparison, evaluation, and reporting of analysis methods. Finally, integrating “multi-omics” datasets, such as from RNA modification profiling or structural probing experiments with CLIP data could provide deeper insights into the factors influencing RNA–RBP interactions. Such data integration could also shed light on the persistent issue of low reproducibility in binding regions observed across different experiments and/or cell lines, offering a more comprehensive understanding of the binding landscape.

In spite of still existing limitations, CLIP and the derived techniques have tremendously advanced the field and have become indispensable to study RNA–protein interactions. In this review, we briefly summarized the major developments, discussed challenges and considered potential future directions in the CLIP field. The continued innovation and refinement of these protocols will be pivotal in advancing our understanding especially of the “brave new world of RNA-binding proteins.”

## Data Availability

The source code and the list of datasets used to generate figure 4 are available in the following github repository: https://github.com/tsekara/CLIP_metaanalysis (https://doi.org/10.5281/zenodo.19361021).
